# Effective Control of Type 2 Diabetes through Antioxidant Defense by Edible Fruits of *Diospyros peregrina*

**DOI:** 10.1093/ecam/nep080

**Published:** 2011-02-20

**Authors:** Saikat Dewanjee, Anup Maiti, Ranabir Sahu, Tarun K. Dua, Vivekananda Mandal

**Affiliations:** Division of Pharmacognosy, Department of Pharmaceutical Technology, Jadavpur University, Kolkata 700032, India

## Abstract

The matured fruits of *Diospyros peregrina* are successfully employed by the traditional healers and local people of costal West Bengal, India for the treatment of diabetes. Present investigation was undertaken to evaluate the role of hydroalcoholic extract of *D. peregrina* (HDP) on type 2 diabetes as well as the augmented oxidative stresses associated with it. Oral administration of HDP at 25, 50 and 100 mg kg^−1^ body weight per day to diabetic rats was found to possess significant dose-dependent hypoglycemic and hypolipidemic activity. The diabetic rats showed lower activities of superoxide dismutase (SOD), catalase (CAT) and reduced glutathione (GSH) content in hepatic and renal tissues as compared to normal rats. The activities of SOD, CAT and GSH were found to be increased in extract-treated diabetic rats in selected tissues. The increased level of lipid peroxidation (thiobarbituric acid reactive substances) in diabetic rats was also found to be reverted back to near normal status in extract-treated groups. Thus it may be concluded that the HDP may produce its hypoglycemic effect through antioxidant defense mechanism.

## 1. Introduction

Diabetes mellitus is a chronic metabolic disorder characterized by elevated blood glucose concentration caused by insulin deficiency, often combined with insulin resistance [1]. By the year 2030, the total number of people worldwide with diabetes mellitus is projected to reach 366 million [2]. Type 2 or non-insulin-dependent diabetes mellitus (NIDDM) accounts for about 90%–95% of all diagnosed cases of diabetes [3]. Hyperglycemia alone does not cause diabetic complications. It is rather the detrimental effect of glucose toxicity due to chronic hyperglycemia mediated and complicated through augmented oxidative stress [4]. Hyperglycemia increases the production of reactive oxygen species (ROS) inside the aortic endothelial cells. ROS-induced activation of protein kinase-C isoforms, increased formation of glucose-derived advanced glycation end products, increased glucose flux through aldose reductase pathways and activation of cytokines are some of the known biochemical mechanisms of hyperglycemia-induced tissue and cell damage [5]. Thus, antioxidant therapy in diabetes may be helpful in relieving symptoms and complications observed in diabetes patients. Many traditional medicinal plants that possess substantial quantity of antioxidant components have been found to be useful against diabetes and its related complications [6, 7]. Hence, there is a huge prospect of development of potential hypoglycemic agent coupled with antioxidant activity from traditional medicinal plants to combat diabetes and its complications [8].

*Diospyros peregrina* Gurke. (Ebenaceae) is a middle-sized tree that grows luxuriantly in the plains of costal region in India. Mature fruits are edible with ethnomedicinal significance as tonic and aphrodisiace [9]. Unripe fruits are astringent, acrid, bitter and oleaginous [10]. Unripe fruits are used for the treatment of diarrhea, dysentery, cholera, mouth ulcer and in wounds [11]. The matured fruits of *D. peregrina* are successfully employed by the traditional healers and local people of costal West Bengal for the treatment of diabetes. The mature fruits of *D. peregrina* contain substantial quantity of phytopolyphenolics, which are known for their important antioxidant activity [12, 13]. The present investigation was undertaken to evaluate the role of hydroalcoholic extract of *D. peregrina* fruit pulps (HDPs) in effective management of type 2 diabetes through antioxidant defense mechanism.

## 2. Methods

### 2.1. Plant Material

Mature fruits of *D. peregrina* were collected in the month of July 2006 from the villages of costal West Bengal, India. The plant was authenticated by H. J. Chowdhury, Joint Director, Botanical Survey of India, Shibpur, Howrah, India. A voucher specimen JU/PT/PC/01/06 was deposited at our laboratory for future reference.

### 2.2. Chemicals

Streptozotocin was procured from SISCO Research Lab, Mumbai, India. Thiobarbituric acid was purchased from Loba Chemie, Mumbai, India. All chemicals and reagents used were of analytical grade.

### 2.3. Preparation of Extract

The fruit pulps were dried in an incubator at 40°C for 1 week, pulverized in an electrical grinder and macerated for 48 h at room temperature with 800 mL of mixture (double distilled water: 99% absolute alcohol; 30% : 70% v/v). The macerate was filtered and concentrated *in vacuo* (at 35°C and 0.8 MPa) and finally lyophilized to yield HDP (7.5% w/w). The total concentration of polyphenolics [14] and flavonoids [15] present in this extract was determined.

### 2.4. Animals

Three-day-old pups of the Wistar albino rats were used for this experiment. Pups were kept with the mother rat in a standard polypropylene cage. Animals were maintained under standard laboratory conditions of temperature (20 ± 2°C), relative humidity (50 ± 15%), 12 h light–dark cycle, standard diet and water *ad libitum*. The principles of Laboratory Animals Care [16] and the instructions given by our institutional animal ethical committee (Registration No: 0367/01/C/CPCSEA) were followed throughout the experiment.

### 2.5. Induction of Diabetes

The neonatal-streptozotocin-diabetic rat model was performed as per the method described by Portha et al. [17]. Rats were injected intraperitoneally with 90 mg kg^−1^ streptozotocin in 0.01 M citrate buffer (pH 4.5) [17, 18]. Twelve weeks after the injection of streptozotocin, the animals exhibiting fasting glucose levels 140–200 mg dL^−1^ were screened as type 2 diabetic and neonatal-streptozotocin-diabetic rats resembling type 2 diabetes in humans [19].

### 2.6. Experimental Design

Animals were divided into six groups of six rats each. Group I: normal rats administered distilled water, 2.0 mL kg^−1^, orally daily for 5 days. Group II: diabetic control rats administered distilled water daily for 5 days. Group III: diabetic rats administered HDP, 25 mg kg^−1^, orally daily for 5 days. Group IV: diabetic rats administered HDP, 50 mg kg^−1^, orally daily for 5 days. Group V: diabetic rats administered HDP, 100 mg kg^−1^, orally daily for 5 days. Group VI: diabetic rats administered standard drug glibenclamide (1 mg kg^−1^, orally) daily for 5 days. Fasting blood glucose levels were estimated on Days 0, 1, 3, 5 with the help of single touch glucometer (Ascensia Entrust, Bayer Health Care, USA). Body weights of experimental rats were measured on Days 1, 3 and 5. After 5 days of treatment, all the rats were anaesthetized and sacrificed by cervical dislocation; livers and kidneys were excised and washed thoroughly to clear off blood. The tissues were immediately transferred to ice-cold saline and homogenized in 0.1N Tris-HCl buffer (pH 7.4). These homogenate tissues were used for the estimation of thiobarbituric acid reactive substances (TBARSs) [20], reduced glutathione (GSH) [21], superoxide dismutase (SOD) [22] and catalase (CAT) [23]. Serum lipids and liver glycogen [24] profiles were also estimated.

### 2.7. Statistical Analysis

Data were statistically calculated by utilizing one-way ANOVA and expressed as mean ± SEM. followed by Dunnett's *t*-test using computerized GraphPad InStat version 3.05, Graph pad software, USA. The values were considered significant when *P *< .05.

## 3. Results

### 3.1. Polyphenolics and Flavonoids Quantity

The level of total polyphenolic compounds was found to be 118.24 mg of pyrocatechol equivalent per gram of dry weight of HDP. Total flavonoids content was found to be 84.45 mg of quercetin equivalent per gram of HDP.

### 3.2. Blood Glucose Status

**Figure 1:**
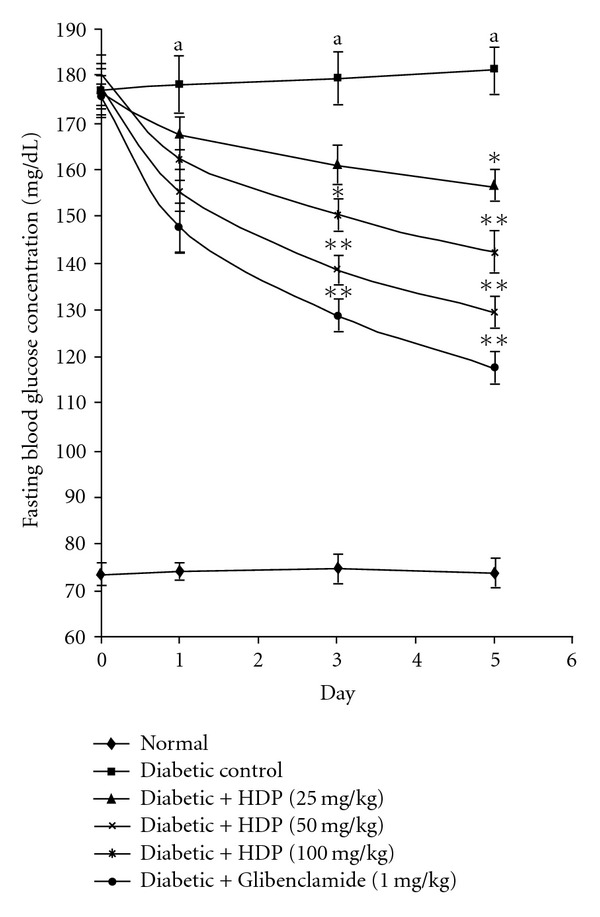
Effect of HDP on fasting plasma glucose level of neonatal-streptozotocin type 2 diabetic rats. Values are expressed as mean + SEM (*n* = 6). ^a^ *P *< .01 compared with normal control group. *P < .05 compared with diabetic control group, **P < .01 compared with diabetic control group.

A significant difference was observed between normal and type 2 diabetic rats (*P *< .01) in fasting blood glucose level. HDP at the doses of 25, 50 and 100 mg kg^−1^ body weight significantly lowered fasting blood glucose level of type 2 diabetic and exhibited maximum reduction of 13.98 (*P *< .05), 23.89 (*P *< .01) and 32.05% (*P *< .01) on Day 5, respectively. The results were compared with standard oral hypoglycemic agent glibenclamide (1 mg kg^−1^), which exhibited maximum reduction of 36.12% (*P *< .01) on Day 5 (Figure 1).

### 3.3. Blood Lipid Status

Significant increase (*P *< .01) in cholesterol and triglycerides levels was observed in diabetic rats when compared with normal control groups. Treatment with HDP significantly lowered the levels of cholesterol and triglyceride in a dose-dependent manner when compared with diabetic control group. The hypolipidemic effect is more at the dose of 100 mg kg^−1^ and results are comparable to that of standard drug glibenclamide. A significant decrease (*P *< .01) in the level of liver glycogen was observed in diabetic rats when compared with normal control groups (Table 1).

### 3.4. Liver Glycogen and Body Weight Status

Oral administration of HDP at the selected doses significantly increases liver glycogen level to its normal level and the result is comparable to that of standard drug glibenclamide (Table 1). HDP treatment also improved body weight profile (statistically insignificant) in type 2 diabetic rats with respect to diabetic control group (Table 2).

### 3.5. Antioxidant Status

**Figure 2:**
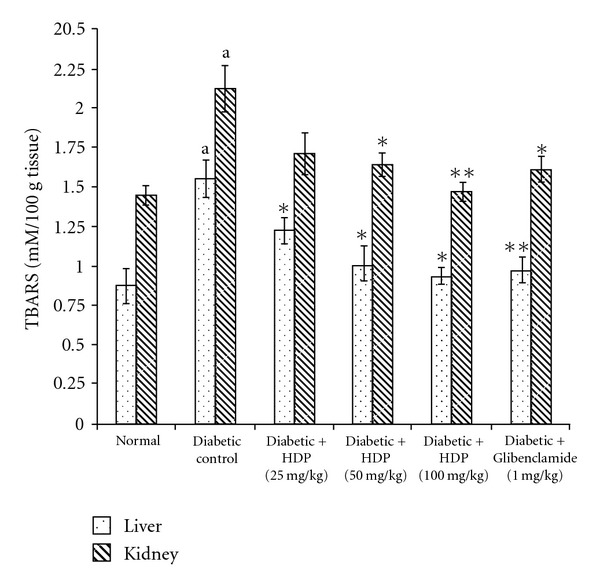
Effect of HDP on TBARS of hepatic and renal tissues of type 2 diabetic rats. Data are mean ± SE of six animals for each group. ^a^ *P *< .01 compared with normal control group. *P < .05 compared with diabetic control group, **P < .01 compared with diabetic control group.

**Figure 3:**
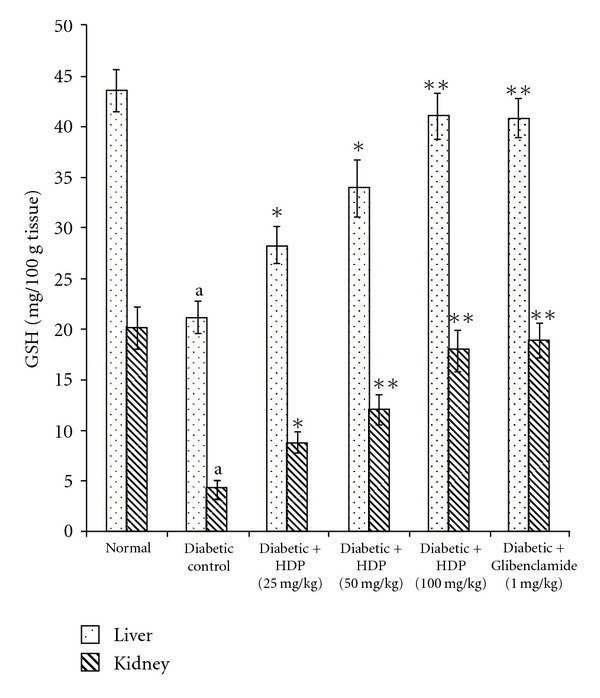
Effect of HDP on GSH of hepatic and renal tissues of type 2 diabetic rats. Data are mean ± SE of six animals for each group. ^a^ *P *< .01 compared with normal control group. *P < .05 compared with diabetic control group, **P < .01 compared with diabetic control group.

**Figure 4:**
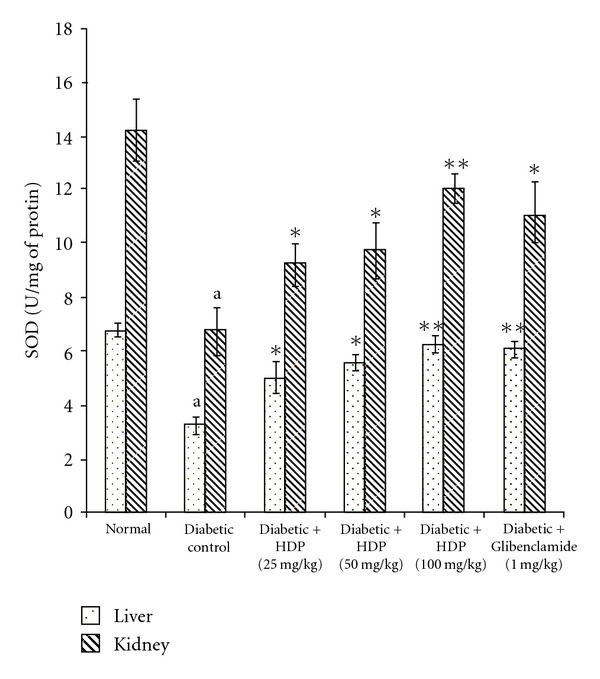
Effect of HDP on SOD of hepatic and renal tissues of type 2 diabetic rats. Data are mean ± SE of six animals for each group. ^a^ *P *< .01 compared with normal control group. *P < .05 compared with diabetic control group, **P < .01 compared with diabetic control group.

**Figure 5:**
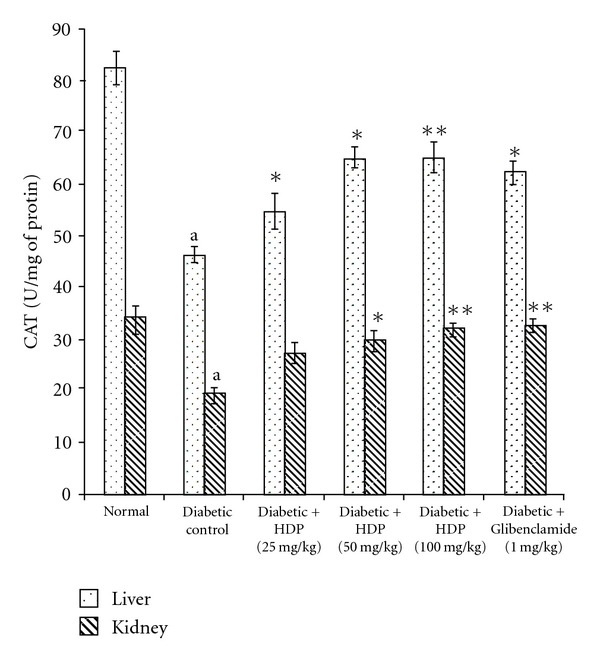
Effect of HDP on CAT of hepatic and renal tissues of type 2 diabetic rats. Data are mean ± SE of six animals for each group. ^a^ *P *< .01 compared with normal control group. *P < .05 compared with diabetic control group, **P < .01 compared with diabetic control group.

The antioxidant effect of the HDP on tissue antioxidant markers was studied. The type 2 diabetic rats showed a significant increase in TBARS in hepatic and renal tissues (*P *< .01). Oral administration of HDP reduced these to normal level (Figure 2). There was a significant reduction (*P *< .01) in GSH in diabetic rats. HDP administration to diabetic rats significantly increased liver and kidney GSH to near normal value (Figure 3). The decreased levels (*P *< .01) of SOD and CAT in diabetic rats were found to be reverted back to near normal status after the treatment of HDP (Figures 4 and 5). The HDP was found to possess antioxidant effect in a dose-dependent manner.

## 4. Discussion

When rats are injected with streptozotocin during the neonatal period, it resembles human type 2 diabetes mellitus with respect to abnormalities in insulin secretory responses [25]. In this model, mild hyperglycemia appears between 2 and 3 months of life, together with a partial deficiency in insulin [26]. In the present study, diabetes control rats exhibited significantly elevated fasting blood glucose, cholesterol and triglyceride levels as compared with normal control rats. Treatment with HDP significantly reduced fasting blood glucose and lipid levels. Maintenance of blood glucose and lipid profile with extract-treated rats vindicates the effectiveness of the extract against experimental type 2 diabetic rats. The significant control of plasma lipid levels suggests that the extract may produce its action by improving insulin secretion [27]. Type 2 diabetic rats exhibited significantly lower level of liver glycogen level, which was significantly reverted back near to the normal status in HDP-treated diabetic groups, may be due to reactivation of the glycogen synthase system by improving insulin secretion. Diabetes is associated with weight loss. The reversal of weight loss in extract-treated diabetic group indicates that the restorative effect of HDP may be by the reversal of gluconeogenesis and glycogenolysis [28].

An increase in hepatic and renal TBARS is an index of enhanced lipid peroxidation in diabetes, which may be due to enhance production or decrease destruction of ROS [27]. Increased TBARS in type 2 diabetic rats significantly lower in both the tissues on HDP treatment. Increased lipid peroxidation in diabetes can be due to enhanced oxidative stress in the cells as a result of depletion of antioxidant scavenger system. GSH is a major endogenous antioxidant, which counteracts free radical-mediated damage. Depletion of liver and kidney GSH levels represents enhanced oxidative stress [28]. Oral administration of HDP significantly elevated GSH level in selected tissues. SOD is an antioxidant enzyme, which reduces superoxide radicals to water and molecular oxygen while CAT reduces hydrogenperoxide [29]. Diminished activity of these antioxidant enzymes result elevation of ROS and ROS-mediated cell destruction. Reduced activities of SOD and CAT in liver and kidney were observed in diabetic rats and these were reverted to near normal status on extract treatment.

**Figure 6:**
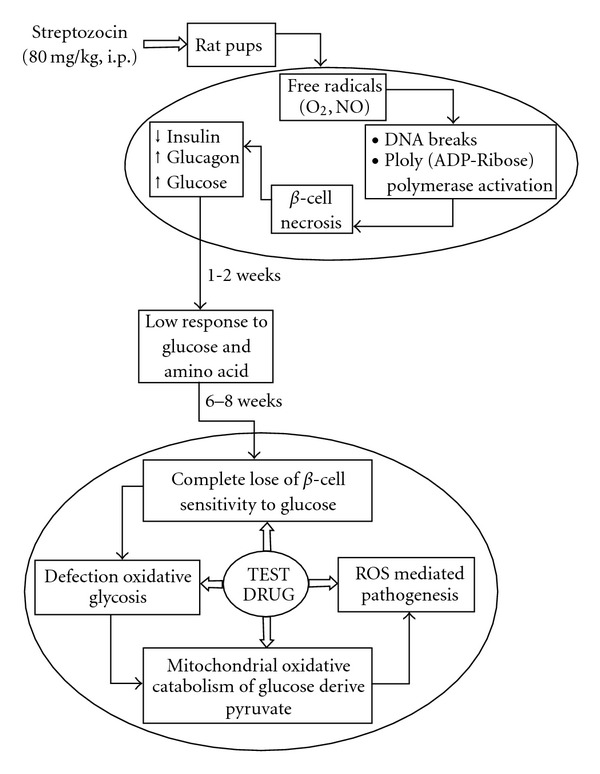
Multimodal therapeutic approach of HDP to counteract with diabetic hyperglycemia and free radical mediated pathogenesis.

Present investigation showed that the HDP possesses considerable hypoglycemic and hypolipidemic activity in type 2 diabetic rats. The HDP also exhibited a profound antioxidant effect in diabetic rats. The multimodal therapeutic approach of HDP has been represented with the help of hypothetical diagram (Figure 6). The antioxidant defense system represents a complex network with interactions, synergy and specific tasks for a given antioxidant [30, 31]. Recent studies showed that majority of the plasma antioxidants are depleted in type 2 diabetes [32]. The depletion of antioxidants in the diabetic condition is a major cause of diabetes-related complications and onset of other disease conditions [33, 34]. Present therapeutic strategies typically attempt to relieve the clinical manifestation of diabetes and its complications [35]. The major challenge in diabetes research is to define not only the cause—effect relationship between various risk factors and complications, but also to comprehend the effects of therapeutic agents that are beneficial in the management of diabetic complications [36]. Nevertheless, the justification for the therapeutic use of antioxidants in cases of diabetes is emerging fast [37]. The quantitative estimation of total polyphenolics and flavonoids confirmed that the HDP contains substantial quantity of polyphenolics and flavonoids, which are the known antioxidant from plant sources [38]. Polyphenolics have also been reported to contribute hypoglycemic activity [39, 40]. So, the polyphenolics-enriched fruits of *D. peregrina* may contribute in effective diabetes management in future.

## Figures and Tables

**Table 1:** Effect of HDP on serum lipids and liver glycogen levels of type 2 diabetic rats.

Group	Cholesterol (mg dl^−1^)	Triglycerides (mg dl^−1^)	Liver glycogen (mg g^−1^)
Normal control	76.83 ± 3.34	78.83 ± 3.12	14.17 ± 1.08
Diabetic control	111.17 ± 4.64*^a^*	112.67 ± 5.91*^a^*	7.45 ± 0.69*^a^*
Diabetic + HDP (25 mg kg^−1^)	91.54 ± 6.12*	92.17 ± 7.03*	12.38 ± 1.01*
Diabetic + HDP (50 mg kg^−1^)	89.24 ± 4.22*	87.67 ± 5.42*	13.35 ± 1.21**
Diabetic + HDP (100 mg kg^−1^)	88.33 ± 4.34**	79.50 ± 4.67**	13.67 ± 1.12**
Diabetic + Glibenclamide (1 mg kg^−1^)	89.67 ± 3.62*	85.50 ± 4.43**	14.02 ± 1.19**

Data are mean ± SE of six animals for each group.

*^a^P *< .01 compared with normal control group.

*P < .05, **P < .01 compared with diabetic control group.

**Table 2:** Effect of HDP fruit on body weight profile of type 2 diabetic rats.

Group	Body weight profile (g)		
	Day 1	Day 3	Day 5
Normal control	164.17 ± 3.01	167.50 ± 2.81	168.33 ± 2.11
Diabetic control	137.50 ± 4.96	135.83 ± 4.36	135.50 ± 3.99
Diabetic + HDP (25 mg kg^−1^)	142.5 ± 3.09	143.33 ± 2.79	144.17 ± 2.82
Diabetic + HDP (50 mg kg^−1^)	140.00 ± 5.77	143.33 ± 6.28	145.83 ± 6.38
Diabetic + HDP (100 mg kg^−1^)	141.67 ± 4.60	140.83 ± 4.55	139.50 ± 5.05
Diabetic + Glibenclamide (1 mg kg^−1^)	140.83 ± 6.23	144.17 ± 6.11	147.50 ± 6.42

Data are mean ± SE of six animals for each group.
